# Boronic prodrug of 4-hydroxytamoxifen is more efficacious than tamoxifen with enhanced bioavailability independent of CYP2D6 status

**DOI:** 10.1186/s12885-015-1621-2

**Published:** 2015-09-09

**Authors:** Qiu Zhong, Changde Zhang, Qiang Zhang, Lucio Miele, Shilong Zheng, Guangdi Wang

**Affiliations:** 1 RCMI Cancer Research Center and Department of Chemistry, Xavier University of Louisiana, 1 Drexel Dr., New Orleans, LA 70125 USA; 2 Department of Genetics and LSU Stanley Scott Cancer Center, LSU Health Sciences Center, 1 Drexel Dr., New Orleans, LA 70112 USA

## Abstract

**Background:**

Poor initial response to tamoxifen due to CYP2D6 polymorphism and adverse side effects are two clinical challenges in tamoxifen therapy. We report the development and preclinical testing of a boronic prodrug to orally deliver 4-OHT at therapeutically effective concentrations but at a fraction of the standard tamoxifen dose.

**Methods:**

A mouse xenograft tumor model was used to investigate the efficacy of ZB497 in comparison with tamoxifen. Pharmacokinetic studies were conducted to evaluate the metabolism and bioavailability of the drug in mice. Drug and metabolites distribution in xenograft tumor tissues was determined by high performance liquid chromatography-tandem mass spectrometry.

**Results:**

The boronic prodrug, ZB497, can not only be efficiently converted to 4-OHT in mice, but also afforded over 30 fold higher plasma concentrations of 4-OHT than in mice given either the same dose of 4-OHT or tamoxifen. Further, ZB497 was more effective than tamoxifen at lowered dosage in inhibiting the growth of xenograft tumors in mice. Consistent with these observations, ZB497 treated mice accumulated over 6 times higher total drug concentrations than tamoxifen treated mice.

**Conclusions:**

Our study demonstrates that ZB497 effectively delivers a markedly increased plasma concentration of 4-OHT in mice. The boronic prodrug was shown to have far superior bioavailability of 4-OHT compared to tamoxifen or 4-OHT administration as measured by the area under the plasma concentration time curve (AUC), plasma peak concentrations, and drug accumulation in tumor tissues. Further, ZB497 proves to be a more efficacious hormone therapy than tamoxifen administered at a reduced dose in mice.

## Background

Tamoxifen remains a safe and effective agent for women diagnosed with ER (+) breast cancer. It is a first-line agent for pre-menopausal breast cancer patients and for women requiring secondary chemoprevention after a DCIS or LCIS diagnosis. It is an option for other ER+ breast cancer patients who do not tolerate the side effects of aromatase inhibitors. Results of the ATLAS trial show that 10-years treatment with tamoxifen further improves long-term survival compared to 5-years treatment [1]. However, the response to tamoxifen shows well-known individual variability [2–8]. Tamoxifen is a pro-drug, which needs to be converted into active metabolites for optimal clinical activity. Cytochrome P450 enzyme CYP2D6 is required to convert tamoxifen into 4-hydroxytamoxifen (4-OHT) and endoxifen [9], both of which are about 100 times more potent than tamoxifen [10, 11]. Genetic polymorphism in CYP2D6 affects the rate of metabolic activation of tamoxifen. This may account for poor initial response to tamoxifen and worse disease outcome after standard therapy. Multiple clinical studies have shown that poor metabolizer (PM) patients tend to have shorter overall survival rate than those who are extensive metabolizers (EM) [4–8]. Existing clinical and laboratory data support the hypothesis that bioavailable 4-OHT or endoxifen could offer improved therapeutic efficacy and potentially lower dose requirements, with reduced adverse effects [12–14]. Indeed, 4-OHT is being developed as a topically applied gel currently in Phase II clinical trials [15–18]. The use of orally available 4-OHT is hampered by its rapid first-pass clearance due to O-glucuronidation [19] and the resulting poor bioavailability compared to oral tamoxifen. Clinical trials utilizing high-dose tamoxifen have been conducted in PM patients in order to increase blood levels of active metabolites. However, this also increases the risk with adverse effects including hot flashes and thrombosis [20].

We have recently developed several boron-derived prodrugs of 4-OHT that demonstrated potent antiestrogenic activities *in vitro* at significantly lower concentrations than tamoxifen [21]. We propose a novel endocrine therapy regimen using ZB497, an orally bioavailable prodrug form of 4-OHT that can be administered at lower doses than standard tamoxifen treatment, thereby not only circumventing the need for CYP2D6 enzyme to catalyze the hydroxylation of tamoxifen or N-desmethyltamoxifen, but also potentially reducing or eliminating side effects by virtue of significantly reduced dosage. In order to further evaluate the prodrug as a potential new option in breast cancer treatment and/or prevention, we conducted in vivo efficacy studies using a well-characterized mouse xenograft model based on the ERα positive MCF-7 breast cancer cells. We determined whether the boron-based 4-OHT prodrug can achieve acceptable in vivo efficacy in an ERα + breast cancer xenograft model as compared to tamoxifen in a dose dependent manner. Pharmacokinetic studies were performed in mice to investigate the metabolism, distribution, and concentration change over time after a single dose of ZB497, in comparison with tamoxifen and 4-OHT. Moreover, tumor tissues from mice were analyzed for drug accumulation after 21 days of treatment of ZB497 or tamoxifen.

## Methods

### Reagents and materials

All reagents, solvents, and analytical standards were purchased from Sigma Aldrich (St. Louis, MO) and Fisher Scientific (Fairfield, NJ). ZB497 were synthesized following the synthetic route described in detail in a previous report [21].

### In vivo efficacy study in mice

Four- to six-week old female ovariectomized Nu/Nu mice were purchased from Charles River Laboratories (Wilmington, MA). The mice were given a period of adaptation in a sterile and pathogen-free environment with phytoestrogen-free food and water ad libitum. MCF-7 cell line was purchased from ATCC (ATCC #HTB-22, Manassas, VA), and routinely cultured in phenol red-free DMEM medium supplemented with 5 % FBS, 4 mM glutamine, 1 mM sodium pyruvate, 100 IU/mL penicillin, 100 μg/mL streptomycin and 0.25 μg/mL amphotericin. The cells were harvested in the exponential growth phase using a PBS/EDTA solution. The animals were injected bilaterally in the mammary fat pad (MFP) with 5 × 10^6^ viable cells suspended in 50 μL sterile PBS mixed with 100 μL Matrigel (reduced growth factor; BD Biosciences, Bedford, MA). 17β- estradiol pellets (0.72 mg, 60-day release; Innovative Research of America, Sarasota, FL) were implanted subcutaneously in the lateral area of the neck using a precision trochar (10 gauge) at the time of cell injection. All procedures in animals were carried out under anesthesia using a mixture of isofluorane and oxygen delivered by mask. Tumors were allowed to form and at day 15 post cell injection mice were randomized into groups of 5 mice each. Mice were treated daily with intraperitoneal (i.p.) injection or oral gavage of either vehicle (1:20 DMSO/PBS for i.p. or 1:10 ethanol/PBS for oral gavage) or a drug for 21 days. For the dose-dependent efficacy study, five groups (5 mice/group) of tumor-bearing nude mice (one control and one group each per dose per drug). Tumor size was measured 3 times weekly using digital calipers. Tumors were surgically removed from sacrificed mice treated with a daily dose of either 1 mg/kg tamoxifen or 1 mg/kg ZB497 by oral gavage for 7 days, weighed, and stored at −80 °C until sample preparation and analysis.

### Pharmacokinetic studies

Female C57BL/6 mice were used for the pharmacokinetic study of ZB497. For intraperitoneal administration of drugs, mice were injected with PBS containing ZB497, tamoxifen, or 4-OHT by adding appropriate amounts of individual stock solutions of the drugs dissolved in DMSO. For oral administration, mice were given oral gavage containing PBS and ethanol-dissolved ZB497, 4-OHT, or tamoxifen at a single dose of 1 mg/kg/mouse. After i.p or oral administration, blood samples were collected from the orbital sinus of the mice at various time points with each group of mice subjected to only one sampling. Mice blood was collected with a capillary into 1.5 mL microcentrifuge tubes containing 0.1 mL of 10 % EDTA anticoagulant. Plasma was then separated from red cells by centrifugation in a refrigerated centrifuge at 4 °C and transferred to a separate tube. The plasma samples were frozen at −80 °C until analysis.

### Analysis of drug concentrations in plasma and tumor tissues

Plasma samples were extracted with chloroform:methanol (2:1) using traditional Folch method for lipid extraction. Methanol (1 mL) and chloroform (2 mL) were added to each plasma sample followed by addition of 5 ng trans-Tamoxifen-^13^C2, ^15^ N to each sample as the internal standard. The mixtures were stored at −20 °C overnight. Next, the samples were sonicated for 5 min and centrifuged with a Thermo Scientific Heraeus Megafuge16 Centrifuge. The top layer was transferred to another test tube. The bottom layer was washed with 1 mL chloroform:methanol (2:1), centrifuged, and the top layer was transferred and combined with the previous top layer. Eight tenth of a milliliter HPLC grade water was added to the extracts. After vortexing, the mixture was centrifuged. The bottom layer was dried out with nitrogen and re-suspended in 100 μL HPLC grade acetonitrile. An aliquot of 10 μL sample was injected onto a Hypersil Gold column (50 × 2.1 mm; particle size 1.9 μm, Thermo Scientific) on a Dionex Ultimate 3000 UPLC system equipped with a TSQ Vantage triple quadrupole mass spectrometer for analysis. A binary mobile phase (A: water with 0.05 % formic acid; B: acetonitrile with 0.05 % formic acid) was used to achieve a gradient of initial 30 % B for 1 min and then to 80 % B at 8 min, to 100 % B at 9 min, and returned to 30 % B for 4 min. The flow rate was controlled at 0.6 mL/min. The settings of HESI source were as follows: spray voltage (3200 volt); vaporizer temperature (365 °C); sheath gas pressure (45 psi); auxiliary gas pressure (10 psi); capillary temperature (330 °C). Nitrogen was used as the sheath gas and auxillary gas. Argon was used as the collision gas.

For determination of drug concentrations in tumor tissues, tumors were initially homogenized in 3 mL chloroform:methanol (2:1 v:v) with a PYREX™ Tenbroeck tissue grinder. The same solvent (1 mL) was used to wash the tissue grinder three times and the washings were combined with the initial homogenized tumor suspension. After adding 5 ng trans-tamoxifen-^13^C2, ^15^ N to each sample as an internal standard, the mixtures were stored at −20 °C overnight. The mixtures were then sonicated for 5 min and centrifuged. The top layer was transferred to another test tube. The bottom layer was washed with 1 mL chloroform:methanol (2:1), centrifuged, and the top layer from this wash was transferred and combined with the previous top layer. After adding water (1.4 mL) to the extract, vortexing and centrifuging, and the bottom layer was dried out with nitrogen and re-suspended in 100 μL HPLC grade acetonitrile for analysis on the HPLC-TSQ instrument under the same conditions as those used for the analysis of the plasma samples.

### Ethical considerations and statistical analysis

All procedures involving the animals were conducted in compliance with State and Federal laws, standards of the U.S. Department of Health and Human Services, and guidelines established by the Institutional Animal Care and Use Committee at Xavier University. All animal experiments were approved by Xavier’s Institutional Animal Care and Use Committee. The facilities and laboratory animals program of Xavier University are accredited by the Association for the Assessment and Accreditation of Laboratory Animal Care. Statistical analyses were performed using Microsoft excel software. Pharmacokinetic data analyses were performed using the PKsoftware [22].

## Results

### The boronic prodrug ZB497 is effective in the inhibition of xenograft tumor growth in mice

**Fig. 1:**
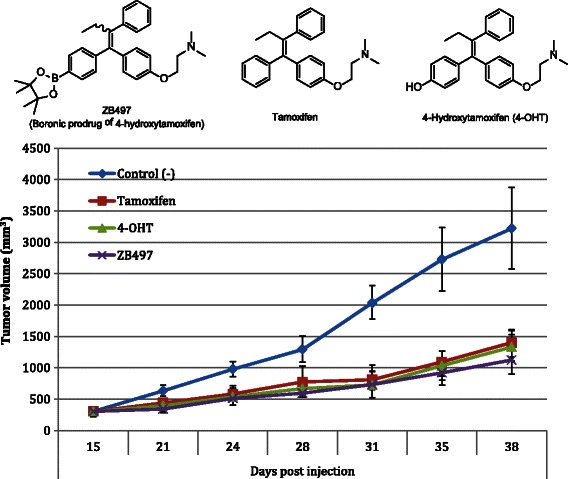
Inhibition of xenograft tumor growth by i.p. administered tamoxifen, 4-OHT, or ZB497. Mice were treated daily with intraperitoneal (i.p.) injection of either vehicle (1:20 DMSO/PBS, tamoxifen, 4-OHT, or ZB497 for 23 days.

We have previously shown that boron-based 4-hydroxytamoxifen prodrugs are active antiestrogenic agents *in vitro*, with potencies exceeding that of tamoxifen in MCF-7 and T47D breast cancer cells the express ERα [21]. To determine if these *in vitro* activities translate to in vivo efficacy, we utilized a nude mouse model in which MCF-7 cells were injected bilaterally into the mammary fat pad (MFP) to form tumor xenografts. Mice were treated daily with intraperitoneal (i.p.) injections of either vehicle (1:5 DMSO/PBS), tamoxifen (1.0 mg/kg), 4-OHT (1.0 mg/kg), or ZB497 (1.0 mg/kg) for 21 days. As shown in Fig. 1, i.p. administered ZB497 was found to inhibit tumor growth in mice as effectively as either tamoxifen or 4-OHT. While statistically insignificant, ZB497 showed a slightly greater degree of efficacy than tamoxifen and 4-OHT treatments.

### Metabolism and pharmacokinetics of ZB497

**Fig. 2:**
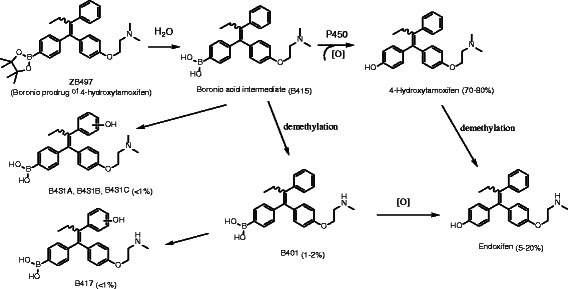
Structures of identified ZB497 metabolic products in mouse blood. A total of eight metabolic products were identified by HPLC-tandem mass spectrometry with approximate range of relative quantities indicated for each structure

As demonstrated in our previous study on breast cancer cells [21], ZB497 was rapidly converted to the boronic acid form of the prodrug before being further transformed to 4-hydroxytamoxifen. To investigate how the prodrug is metabolized and distributed in mice, we measured plasma concentrations of the prodrug and its metabolites over a course of 7 days upon a single dose by either i.p. injection or oral gavage. As illustrated in Fig. 2, a total of 8 metabolic products of ZB497 were identified through the analysis of mouse plasma. ZB497 is first hydrolyzed to B415, the boronic acid, followed by a facile conversion to 4-OHT (70–80 %) and endoxifen (5–20 %), two active metabolites of tamoxifen. The predominant biotransformation route is an oxidative deboronation catalyzed by P450 enzymes in the presence of reactive oxygen species (ROS) [23]. For example, the presence of hydrogen peroxide may contribute to the oxidative cleavage of the boron-aryl carbon bond. Several minor metabolites make up approximately 1–3 % of the total, including B401 (1-2 %), a precursor of endoxifen and product of demethylation of B415, B417 (<1 %), a product of hydroxylation of B401, and B413A, B413B, and B413C (<1 % total), isomeric mixture of metabolites resulting from hydroxylation of B415.

**Fig. 3:**
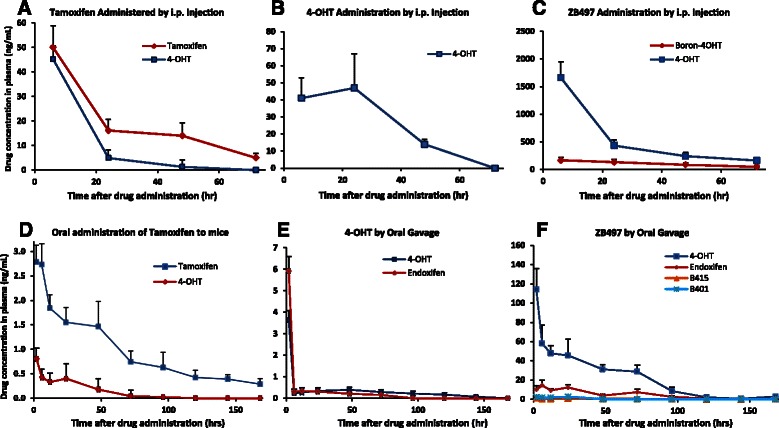
Pharmacokinetics of tamoxifen, 4-hydroxytamoxifen, and ZB497 in mice after i.p. administration (**a**-**c**), or oral administration (**d**-**f**) at a single dose of 1 mg/kg. For i.p. administration of drugs, mice were injected with PBS containing ZB497, tamoxifen, or 4-OHT by adding appropriate amounts of individual stock solutions of the drugs dissolved in DMSO. For oral administration, mice were given oral gavage containing PBS and ethanol-dissolved ZB497, tamoxifen, or 4-OHT

To determine drug concentrations in mouse plasma, B497, 4-OHT, or tamoxifen were dissolved in DMSO, diluted in PBS, and injected intraperitoneally into the mice at a single dose of 1 mg/kg. Blood samples were then taken from mice at various time intervals for analysis of drugs and related compounds by UPLC-MS/MS. The results are summarized in Fig. 3a-c. In the case of tamoxifen i.p. administration, the peak concentration of tamoxifen and 4-OHT were observed in the 6-h blood samples at 50 ng/mL and 45 ng/mL, respectively. Both tamoxifen and 4-OHT concentrations decreased rapidly to non-detectable levels on Day 3 (72 h, Fig. 3a). In mice injected with 4-OHT, the only detected and quantifiable compound in blood was 4-OHT (Fig. 3b) which varied from 47 ng/mL at 24 h to below detection limit at 72 h. At the same dose, however, ZB497 resulted in a peak concentration of over 1600 ng/mL, nearly 40-fold increase in plasma concentration of 4-OHT over the administration of 4-OHT or tamoxifen (Fig. 3c). These results were at once encouraging and surprising. The in vivo PK data not only confirmed the hypothesis that the boronic prodrug can be efficiently converted to 4-OHT, the desired active form of the drug, in animals, but also far exceeded our expectation in terms of vastly improved bioavailability, namely, a 40-fold increase of peak plasma concentrations of 4-OHT.

Mindful of the potential clinical applications for ZB497, we next examined the pharmacokinetics of the boron prodrug when orally administered to mice. In this experiment, mice were given oral gavage containing PBS and ethanol-dissolved tamoxifen, 4-OHT, or ZB497, at a single dose of 1 mg/kg. Blood samples were taken from mice at various time points ranging from 2 h to 7 days after the single dose. As shown in Fig. 3d, a single oral dose of tamoxifen resulted in a peak tamoxifen and 4-OHT concentration of 2.8 and 0.8 ng/mL, respectively, both reached at 2 h. In mice given 4-OHT, the peak concentration of 4-OHT after its oral administration was reached at 2 h at 3.6 ng/mL, but rapidly decreased to below 0.4 ng/mL after 6 h (Fig. 3e). In contrast, when mice were orally administered with ZB497 (Fig. 3f), the peak 4-OHT concentration reached a high level of 114 ng/mL, a value that is 32 times higher than in 4-OHT administration, and over 140 times higher than the 4-OHT concentration from tamoxifen administration. Thus, oral administration of ZB497 also afforded a dramatically increased 4-OHT level in systemic circulation.

The pharmacokinetic parameters were calculated using an excel-based PK software [22] to obtain the t_1/2_ and area under curve (AUC) values for all three drugs. As shown in Table 1, tamoxifen afforded an AUC of 160.6 ng/mL*h for tamoxifen and 21.3 ng/mL*h for 4-OHT, compared to 61.5 for 4-OHT in mice given oral gavage of 4-OHT directly. While tamoxifen administration did not yield quantifiable endoxifen levels, 4-OHT orally given to mice generated an endoxifen concentration comparable to 4-OHT, with an AUC value of 61.2 ng/mL*h. With ZB497 administration, both 4-OHT and endoxifen were measured at dramatically higher levels in plasma with AUC values reaching 3777.7 ng/mL*h for 4-OHT, and 767.4 ng/mL*h for endoxifen. It is well known that tamoxifen metabolism in mice differs significantly from that in humans in terms of the plasma level of 4-hydroxytamoxifen and endoxifen. In mice treated with tamoxifen, the relative concentration of 4-OHT is much higher than that detected in humans whereas the endoxifen level is lower than in humans [23]. Thus, the ratio of 4-OHT from ZB497 to that from tamoxifen may not reflect what might be observed in humans.

**Table 1:** Pharmacokinetic parameters for tamoxifen, 4-OHT, and ZB497 following single dose, oral administration

	Tamoxifen (1 mg/kg)	4-hydroxytamoxifen (1 mg/kg)	ZB497 (1 mg/kg)
*t*_max_ (hrs)	2 (Tam)	2 (4-OHT)	2 (4-OHT)
	2 (4-OHT)	2 (Endoxifen)	6 (Endoxifen)
*t*_1/2_ (hrs)	32.5 (Tam)	31.7 (4-OHT)	39.5 (4-OHT)
	22.6 (4-OHT)	19.5 (Endoxifen)	36.5 (Endoxifen)
C_max_ (ng/mL)	2.78 (Tam)	3.6 (4-OHT)	114.0 (4-OHT)
	0.80 (4-OHT)	5.9 (Endoxifen)	14.4 (Endoxifen)
AUC (ng/mL*h)	160.6 (Tam)	61.5 (4-OHT)	3777.7 (4-OHT)
	21.3 (4-OHT)	61.2 (Endoxifen)	767.4 (Endoxifen)

### ZB497 is more effective than tamoxifen at lowered dose

**Fig. 4:**
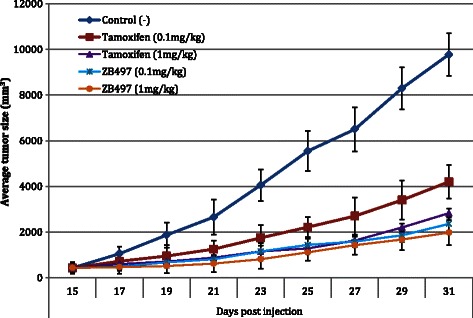
Dose-dependent inhibition of tumor growth in mice that were given oral gavage of vehicle, tamoxifen, or ZB497 at two different doses. Five groups (5 mice/group) of mice bearing MCF-7 xenograft tumors were treated daily with oral gavage of either vehicle (1:10 ethanol/PBS) or a drug at 0.1 mg/kg or 1 mg/kg for 21 days

It would follow that such dramatic enhancement in plasma concentration of an active drug must have therapeutic consequences. To investigate the effects of increased bioavaibility of 4-OHT enabled by the prodrug ZB497 on therapeutic efficacy, mice bearing MCF-7 xenograft tumors were given either tamoxifen or ZB497 at two oral dose levels, 1 mg/kg and 0.1 mg/kg (Fig. 4). At the higher dose of 1 mg/kg, ZB497 exhibited slightly better efficacy than tamoxifen in inhibiting tumor growth, albeit not statistically significant at earlier data points. Still, on day 31, 1 mg/kg of ZB497 group had an average tumor size of 1982 mm^3^, compared to the 1 mg/kg tamoxifen group which had an average tumor size of 2829 mm^3^. When the oral dose was lowered to 0.1 mg/kg, the mice group given tamoxifen showed significantly decreased efficacy in tumor growth inhibition, with the average tumor size reaching 4205 mm^3^ at end of treatment (p < 0.05 compared to 1 mg/kg tamoxifen treatment). In contrast, ZB497 at 0.1 mg/kg dose remained at least as effective as a 10-times higher dose of tamoxifen (1 mg/kg) with the final average tumor size measured at 2364 mm^3^ (p < 0.05 compared to 0.1 mg/kg tamoxifen treatment). This finding provided crucial evidence that the enhanced plasma drug level afforded by ZB497 can be translated to increased therapeutic efficacy.

### ZB497 treatment resulted in higher drug concentrations in tumor

As a cancer treatment regimen, relatively high drug concentration in tumor tissue compared to non-target tissues is advantageous for many reasons, including enhanced efficacy and reduced side effects. When compared to tamoxifen, ZB497 and related metabolites were found to have significantly higher concentrations in mouse blood, which may be the main reason why ZB497 remained therapeutically effective at lower doses than tamoxifen. To determine if indeed higher blood concentration of the drug and its metabolites results in enrichment in tumor tissues, we analyzed xenograft tumor tissues for all measurable drug concentrations.

**Fig. 5:**
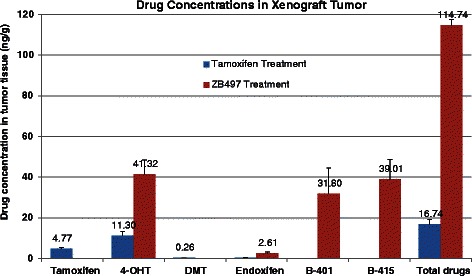
Comparison of individual and total drug and metabolite concentrations in tumor tissues from mice treated with ZB497 or tamoxifen. Tamoxifen, 4-OHT, and desmethyltamoxifen (DMT) were detected and quantified in tumor tissues from tamoxifen treated mice. In tumor tissues from ZB497-treated mice, 4-OHT, endoxifen, B401 and B415 were detected and quantified

Tumor samples were analyzed for tamoxifen, desmethyltamoxifen (DMT), 4-hydroxytamoxifen, endoxifen, and all boron-containing forms of the drug. Figure 5 shows the concentration of each of these drug forms measured in excised tumors. In tumors from tamoxifen-treated mice, the average concentration of tamoxifen, 4-OHT, DMT, and endoxifen were 4.77, 11.30, 0.26 and 0.4 ng/g, respectively, with the total drug concentration in tumor tissues reaching 16.74 ng/g. In ZB497-treated mice, the most abundant drug form was 4-OHT at 41.32 ng/g, nearly 4 times higher than in the tamoxifen-treated group. Endoxifen level in ZB497-treated mice was measured at 2. 61 ng/g, a 6-fold increase compared to the tamoxifen-treated mice. Moreover, B415, the boronic acid form of the prodrug ZB497 reached 39.01 ng/g and the demethylated boronic acid (B401) was measured to be 31.80 ng/g, in contrast to the metabolite distribution in mouse blood, where the boron-containing drug forms were significantly less abundant. The total concentration of all quantifiable drugs after repeated tamoxifen doses is 16.74 ng/g, whereas the total drug concentration resulting from repeated ZB497 doses at 1 mg/kg reached 114.74 ng/g, demonstrating the superior overall bioavailability of the boronic prodrug of 4-OHT in tumor. Importantly, as evidenced in the previous *in vitro* pharmacology study [21], the boronic prodrug forms, including B415, are also potent antiestrogenic agents, thus the accumulation of the boronic prodrug forms in tumor can have two-fold benefits for hormone therapy. First, they contribute to the overall active drug concentration in tumor cells. Second, they serve as a reservoir to continuously release the active tamoxifen metabolites, 4-OHT and endoxifen as a result of oxidative deboronation in situ, by reactive oxygen species such as hydrogen peroxide within tumor cells.

## Discussion

Tamoxifen is a prodrug and its full therapeutic effect depends on the biotransformation of tamoxifen into its active metabolites, 4-OHT and endoxifen. Thus the use of 4-OHT as an agent for endocrine therapy is attractive in that not only it is 100-fold more potent as an antiestrogen than tamoxifen but also 4-OHT has well-known pharmacological and toxicological profiles. It can circumvent the problem associated with deficient CYP2D6 metabolism and potentially lower the dosage requirement owing to its inherent high potency. The problem lies in 4-OHT’s rapid first-pass metabolism, and the poor bioavailability of 4-OHT when used directly via oral administration.

By introducing a cleavable boron-aryl carbon bond to replace the hydroxyl group in 4-OHT, we have designed and tested a boronic prodrug, ZB497 for its ability to overcome the poor bioavailability of 4-OHT. Our study has demonstrated that ZB497 can not only effectively deliver the desired form of the drug, 4-OHT in an animal model, but also at a much higher plasma concentration. The facile oxidative deboronation of the prodrug under physiological conditions is likely facilitated by the presence of P450 enzymes and/or reactive oxygen species [24]. In mouse blood after oral or intraperitoneal administration of ZB497, the predominant drug form is 4-OHT, constituting about 75-85 % of total drug concentration in blood. In addition, compared to tamoxifen or 4-OHT treatment, ZB497 also afforded higher levels of endoxifen in plasma, independent of CYP2D6 status. Such guaranteed delivery of 4-OHT can effectively overcome differences in therapeutic efficacy due to variations in patient metabolism.

But more importantly, the boronic prodrug yielded an astonishingly higher concentration of 4-OHT in systemic circulation than was expected, lending itself to an equally enhanced bioavailability that would have favorable clinical implications. The 30- to 40-fold increase in plasma concentration of 4-OHT upon i.p. or oral administration of ZB497, compared to 4-OHT administration at equal dosage, strongly suggests that the boronic structure does more than just delivering the desired 4-OHT form while preventing its rapid clearance from the body via glucuronidation. Through a mechanism not yet fully accounted for, the boronic structure must be responsible for enabling the remarkable enrichment in plasma concentration of 4-OHT as measured in our pharmacokinetic studies. From the metabolism data both *in vitro* [21] and in vivo in the current study, we know that the predominant form of ZB497 is B415, the boronic acid form of the prodrug. It is well known that boronic acids can form reversible complexes with 1,2 and 1,3 diol groups common in sugar molecules and glycoproteins [25, 26], which upon hydration would release the boronic acids. We hypothesize that such reversible, covalent complexes between boronic acid and a diol group may facilitate the enrichment in plasma due to the abundance of molecules containing the diol groups. Moreover, such complexes may serve as a reservoir to release the boronic acid as the prodrug undergoes deboronation to be converted to 4-OHT. Indeed, this unique property of boronic acid has recently been exploited for an enhanced delivery of insulin for treatment of diabetes [27]. Consequently, in addition to markedly greater plasma concentration, the clearance time of 4-OHT from ZB497 (t_1/2_ = 39.5 hrs) is significantly prolonged compared to direct administration of 4-OHT (t_1/2_ = 31.7 h) or tamoxifen (t_1/2_ for 4-OHT = 22.6 h).

As an immediate result of increased plasma 4-OHT concentration, we found that xenograft tumor tissues also have significantly higher drug accumulation. We also noted that in tumor tissues, the percentage of boron-containing drugs was significantly higher than in plasma after repeated doses of ZB497, suggesting a preferential uptake of the boronic acid form of the prodrug by cancer cells. Nevertheless, 4-OHT concentration in tumor tissues from ZB497-treated mice was still 4 times higher than found in mice treated with tamoxifen.

We believe that enhanced overall bioavailability of 4-OHT conferred by ZB497, as evidenced in the over 30 fold increase in peak plasma concentration and the 8-fold increase in total drug accumulation in tumor tissues culminated in ZB497’s superior in vivo efficacy. At the dosage lowered to 1/10 of a milligram per kilogram, ZB497 remained therapeutically effective in inhibiting xenograft tumor growth, whereas tamoxifen largely lost its efficacy for tumor inhibition at this dose. On the other hand, at the higher dosage of 1 mg/kg, the efficacy difference between ZB497 and tamoxifen was not as prominent. In addition, the degree of tumor growth inhibition by ZB497 at two doses was also similar, and not proportional to the 10-fold difference in dosage. We speculate that the maximum therapeutically effective drug concentration in systemic circulation may have been reached by ZB497 at 0.1 mg/kg and by tamoxifen at 1 mg/kg. Thus, further increasing the dosage of ZB497 to 1 mg/kg may not lead to proportional increase in efficacy in mice.

As demonstrated in the efficient metabolic conversion of ZB497 to 4-hydroxytamoxifen in mice, the prodrug aims to deliver a therapeutically effective dose of the active drug form, 4-OHT. Therefore, it is conceivable that the main possible side effects of the prodrug would be associated with 4-OHT, which is a SERM that behaves similarly as tamoxifen with up to 100-fold greater potency than tamoxifen. It is unknown if 4-OHT may exert 100-fold greater side effects if given at the same dose, but at a fraction of the dose that may be required to reach a therapeutic level, the side effects may not exceed those of tamoxifen. Moreover, because use of ZB497 will eliminate tamoxifen and other related metabolites in systemic circulation, side effects due to these substances may also be eliminated.

Each dose of one mg of ZB497 will generate 0.23 mg of pinacol and 0.12 mg of boric acid. At these low levels, both pinacol and boric acid are not known to be toxic to humans. The LD50 of pinacol in mouse is 3,380 mg/kg oral dose [28] and the LD50 of boric acid in rat is 2660 mg/kg [29]. Notably, the mean daily intakes of boron from food and beverages alone for male and female adults are 1.28 and 1.00 mg respectively [30]. These data suggest that side effects due to the boronic structure of ZB497 may be minimal to nonexistent.

## Conclusions

We have discovered that the boronic prodrug of 4-OHT offers potential solutions to two clinical problems facing tamoxifen therapy. First, patients with deficient CYP2D6 enzymatic function due to genetic polymorphism or due to drug-drug or drug-supplement interaction may not benefit fully from tamoxifen therapy. ZB497 has been shown to deliver the active ingredient, 4-OHT to the systemic circulation in an animal model. Second, persistent side effects such as hot flashes have contributed to low patient compliance with long-term treatment regimens, raising the risk of cancer recurrence. ZB497 is shown to have a unique advantage in that it can reach therapeutically effective concentration of 4-OHT at a fraction of the dose that would be required if tamoxifen or 4-OHT is administered. Lower dosage in the clinic could translate to reduced side effects, potentially increasing patient compliance and lower the risk of cancer recurrence.

## Abbreviations

CYP2D6: Cytochrome P450 2D6
ER: Estrogen receptor
4-OHT: 4-hydroxytamoxifen
AUC: Area under time curve
DCIS: Ductal carcinoma in situ
LCIS: Lobular carcinoma in situ
ATLAS: Adjuvant Tamoxifen Longer Against Shorter
EM: Extensive metabolizer
PM: Poor metabolizer
DMSO: Dimethyl sulfoxide
ROS: Reactive oxygen species
PK: Pharmacokinetics
DMT: Desmethyltamoxifen
